# Supplementary material for: Unveiling the Bioactive Potential of the Invasive Jellyfish Phyllorhiza punctata Through Integrative Transcriptomic and Proteomic Analyses
Source: Biomolecules. 2025 Aug 4;15(8):1121. doi: 10.3390/biom15081121 (PMC12383608; doi:10.3390/biom15081121)
Supplement: Supplementary file 1 [file biomolecules-15-01121-s001.zip › Suplementary Material/FileS3/PEPFOLD4/XP_065051484_1199/clusters.html]

 PEP-FOLD clusters


Download archive of all models  here   

|  |  |  |
| --- | --- | --- |
| # | file | sOPEP |
| # cluster 1 - Size: 23 |  |  |
|  | XP\_065051484\_1199-model1 | -8.76532 |
|  | XP\_065051484\_1199-model1.1 | -8.47232 |
|  | XP\_065051484\_1199-model1.2 | -8.47232 |
|  | XP\_065051484\_1199-model1.3 | -8.20463 |
|  | XP\_065051484\_1199-model1.4 | -8.20463 |
|  | XP\_065051484\_1199-model1.5 | -8.19484 |
|  | XP\_065051484\_1199-model1.6 | -8.17469 |
|  | XP\_065051484\_1199-model1.7 | -8.14379 |
|  | XP\_065051484\_1199-model1.8 | -8.09601 |
|  | XP\_065051484\_1199-model1.9 | -8.07318 |
|  | XP\_065051484\_1199-model1.10 | -8.06183 |
|  | XP\_065051484\_1199-model1.11 | -8.05953 |
|  | XP\_065051484\_1199-model1.12 | -7.97933 |
|  | XP\_065051484\_1199-model1.13 | -7.9718 |
|  | XP\_065051484\_1199-model1.14 | -7.89357 |
|  | XP\_065051484\_1199-model1.15 | -7.85724 |
|  | XP\_065051484\_1199-model1.16 | -7.75547 |
|  | XP\_065051484\_1199-model1.17 | -7.72384 |
|  | XP\_065051484\_1199-model1.18 | -7.47183 |
|  | XP\_065051484\_1199-model1.19 | -7.41374 |
|  | XP\_065051484\_1199-model1.20 | -7.38764 |
|  | XP\_065051484\_1199-model1.21 | -7.30779 |
|  | XP\_065051484\_1199-model1.22 | -6.46398 |
| # cluster 2 - Size: 15 |  |  |
|  | XP\_065051484\_1199-model2 | -8.5941 |
|  | XP\_065051484\_1199-model2.1 | -8.5941 |
|  | XP\_065051484\_1199-model2.2 | -8.58174 |
|  | XP\_065051484\_1199-model2.3 | -8.23657 |
|  | XP\_065051484\_1199-model2.4 | -8.056 |
|  | XP\_065051484\_1199-model2.5 | -7.94753 |
|  | XP\_065051484\_1199-model2.6 | -7.6915 |
|  | XP\_065051484\_1199-model2.7 | -7.2765 |
|  | XP\_065051484\_1199-model2.8 | -7.12053 |
|  | XP\_065051484\_1199-model2.9 | -6.84058 |
|  | XP\_065051484\_1199-model2.10 | -6.67738 |
|  | XP\_065051484\_1199-model2.11 | -6.22718 |
|  | XP\_065051484\_1199-model2.12 | -6.0399 |
|  | XP\_065051484\_1199-model2.13 | -5.88835 |
|  | XP\_065051484\_1199-model2.14 | -5.83842 |
| # cluster 3 - Size: 14 |  |  |
|  | XP\_065051484\_1199-model3 | -8.48648 |
|  | XP\_065051484\_1199-model3.1 | -8.08276 |
|  | XP\_065051484\_1199-model3.2 | -8.08276 |
|  | XP\_065051484\_1199-model3.3 | -8.01063 |
|  | XP\_065051484\_1199-model3.4 | -7.65735 |
|  | XP\_065051484\_1199-model3.5 | -7.62967 |
|  | XP\_065051484\_1199-model3.6 | -7.62204 |
|  | XP\_065051484\_1199-model3.7 | -7.60235 |
|  | XP\_065051484\_1199-model3.8 | -7.60235 |
|  | XP\_065051484\_1199-model3.9 | -7.55376 |
|  | XP\_065051484\_1199-model3.10 | -7.43109 |
|  | XP\_065051484\_1199-model3.11 | -7.23694 |
|  | XP\_065051484\_1199-model3.12 | -6.67263 |
|  | XP\_065051484\_1199-model3.13 | -6.50176 |
| # cluster 4 - Size: 9 |  |  |
|  | XP\_065051484\_1199-model4 | -8.44379 |
|  | XP\_065051484\_1199-model4.1 | -8.29 |
|  | XP\_065051484\_1199-model4.2 | -7.76853 |
|  | XP\_065051484\_1199-model4.3 | -7.61345 |
|  | XP\_065051484\_1199-model4.4 | -7.57287 |
|  | XP\_065051484\_1199-model4.5 | -7.27574 |
|  | XP\_065051484\_1199-model4.6 | -7.11 |
|  | XP\_065051484\_1199-model4.7 | -7.08149 |
|  | XP\_065051484\_1199-model4.8 | -6.90174 |
| # cluster 5 - Size: 5 |  |  |
|  | XP\_065051484\_1199-model5 | -8.35772 |
|  | XP\_065051484\_1199-model5.1 | -7.62399 |
|  | XP\_065051484\_1199-model5.2 | -7.14494 |
|  | XP\_065051484\_1199-model5.3 | -7.14337 |
|  | XP\_065051484\_1199-model5.4 | -6.28514 |
| # cluster 6 - Size: 11 |  |  |
|  | XP\_065051484\_1199-model6 | -8.09185 |
|  | XP\_065051484\_1199-model6.1 | -8.05414 |
|  | XP\_065051484\_1199-model6.2 | -7.98602 |
|  | XP\_065051484\_1199-model6.3 | -7.68611 |
|  | XP\_065051484\_1199-model6.4 | -7.67858 |
|  | XP\_065051484\_1199-model6.5 | -7.61053 |
|  | XP\_065051484\_1199-model6.6 | -7.52404 |
|  | XP\_065051484\_1199-model6.7 | -7.13617 |
|  | XP\_065051484\_1199-model6.8 | -6.84282 |
|  | XP\_065051484\_1199-model6.9 | -6.5995 |
|  | XP\_065051484\_1199-model6.10 | -6.49742 |
| # cluster 7 - Size: 6 |  |  |
|  | XP\_065051484\_1199-model7 | -7.85733 |
|  | XP\_065051484\_1199-model7.1 | -7.52182 |
|  | XP\_065051484\_1199-model7.2 | -7.40058 |
|  | XP\_065051484\_1199-model7.3 | -7.40058 |
|  | XP\_065051484\_1199-model7.4 | -7.18695 |
|  | XP\_065051484\_1199-model7.5 | -6.23184 |
| # cluster 8 - Size: 5 |  |  |
|  | XP\_065051484\_1199-model8 | -7.29751 |
|  | XP\_065051484\_1199-model8.1 | -7.17074 |
|  | XP\_065051484\_1199-model8.2 | -7.03357 |
|  | XP\_065051484\_1199-model8.3 | -6.9363 |
|  | XP\_065051484\_1199-model8.4 | -6.43335 |
| # cluster 9 - Size: 5 |  |  |
|  | XP\_065051484\_1199-model9 | -7.16352 |
|  | XP\_065051484\_1199-model9.1 | -7.09344 |
|  | XP\_065051484\_1199-model9.2 | -6.9558 |
|  | XP\_065051484\_1199-model9.3 | -6.91698 |
|  | XP\_065051484\_1199-model9.4 | -6.34517 |
| # cluster 10 - Size: 3 |  |  |
|  | XP\_065051484\_1199-model10 | -6.7906 |
|  | XP\_065051484\_1199-model10.1 | -6.51757 |
|  | XP\_065051484\_1199-model10.2 | -6.17936 |
| # cluster 11 - Size: 1 |  |  |
|  | XP\_065051484\_1199-model11 | -6.78658 |
| # cluster 12 - Size: 1 |  |  |
|  | XP\_065051484\_1199-model12 | -6.55191 |
| # cluster 13 - Size: 2 |  |  |
|  | XP\_065051484\_1199-model13 | -6.24046 |
|  | XP\_065051484\_1199-model13.1 | -6.08742 |
|  |  |  |
